# Impact of mismatch repair or microsatellite status on the prognosis and efficacy to chemotherapy in metastatic colorectal cancer patients: A bi-institutional, propensity score-matched study

**DOI:** 10.7150/jca.50285

**Published:** 2022-07-11

**Authors:** Yi-Chen Yao, Ying Jin, Xue-Fen Lei, Zi-Xian Wang, Dong-Sheng Zhang, Feng-Hua Wang, Yu-Hong Li, Rui-Hua Xu, Feng Wang

**Affiliations:** 1 State Key Laboratory of Oncology in South China, Collaborative Innovation Center for Cancer Medicine, Sun Yat-sen University Cancer Center, Guangzhou 510060, P. R. China.; 2 Research Unit of Precision Diagnosis and Treatment for Gastrointestinal Cancer, Chinese Academy of Medical Sciences, Guangzhou 510060, P. R. China.; 3 Department of Medical Oncology, The Second Affiliated Hospital of Kunming Medical University, 374 Dianmian Avenue Wuhua district, Kunming, 650101, P. R. China.

**Keywords:** Colorectal cancer, Deficient mismatch repair, Microsatellite instability, Chemotherapy, Prognosis

## Abstract

**Background:** Deficient mismatch repair (dMMR) or the microsatellite instability (MSI) phenotype occupied approximately 15-18% of CRC patients. Previous studies showed that dMMR/MSI status is a favorable prognostic factor for stage II/III CRC patients. For metastatic colorectal cancer (mCRC) patients, only 5% of patients have the dMMR/MSI-H phenotype. The relationship between dMMR/MSI, chemosensitivity and survival in mCRC patients of real-world is still not clear.

**Materials and methods:** In this study, we enrolled 77 dMMR/MSI-H mCRC patients and compared their clinicopathological characteristics with those of 510 proficient MMR (pMMR) or microsatellite stable (MSS) mCRC patients. With propensity score matching (PSM) analysis, we further compared the chemosensitivity and survival of dMMR/MSI-H mCRC patients with pMMR/MSS patients. We also analyzed the efficacy of different chemotherapy and target therapy in the dMMR/MSI-H population.

**Results:** In PSM cohort, the objective response rate (ORR) of mCRC patients with dMMR/MSI-H undergoing first-line palliative chemotherapy was 35.2%, which was similar with patients with pMMR/MSS (35.4%, *p* = 1.00). The median progression-free survival (PFS) of first-line chemotherapy was significantly different (dMMR/MSI-H vs pMMR/MSS = 7.4 months vs 10.2 months; HR = 0.74; 95%CI, 0.57-0.98; *p* = 0.03). Overall survival (OS) of patients did not significantly differ by status (dMMR/MSI-H vs pMMR/MSS = 40.0 months vs 41.3 months; HR = 1.09; 95%CI, 0.74-1.59; *p* = 0.68). For second-line palliative chemotherapy, there was no difference in ORR (*p* = 0.53) or in PFS (HR = 0.88; 95%CI, 0.59-1.33; *p* = 0.56) between dMMR/MSI-H and pMMR/MSS tumors. We also found that in the overall cohort, the ORR of patients who received oxaliplatin-based and irinotecan-based chemotherapy were 28.8% and 54.5%, respectively, which were not significantly different (*p* = 0.16). Our results also showed that the use of bevacizumab could lead to a significantly higher ORR in dMMR/MSI-H mCRC patients compared to chemotherapy alone (55.0% vs 22.2%;* p* = 0.02), whereas cetuximab could not.

**Conclusion:** The dMMR/MSI-H is not a prognostic factor for mCRC patients but is correlated with shorter PFS to first-line palliative chemotherapy.

## Introduction

Colorectal cancer (CRC) is the one of most common cancer in the world 1. Loss of function of DNA mismatch repair (MMR) is an important mechanism of CRC development 2. Mutation or modification of MMR genes result in MMR protein deficient (dMMR) and microsatellite instability (MSI). It has been reported that the dMMR or MSI high (MSI-H) phenotype is present in approximately 15-18% of CRC patients 3. Most dMMR/MSI-H tumors are sporadic CRC, and only approximately 3% of dMMR/MSI-H tumors are Lynch syndrome (LS) or hereditary nonpolyposis colorectal carcinoma (HNPCC) 4, 5.

The dMMR/MSI-H status was reported to be a predictive marker for adjuvant chemotherapy. Multiple retrospective studies showed that dMMR/MSI-H is correlated with a favorable prognosis in stage II/III CRC 6-11. Previous studies suggested that dMMR/MSI status may be a predictive marker of decreased benefit form adjuvant monotherapy of 5-fluorouracil (5-FU) in patients with stage II disease, but not in those with stage III disease 7, 8, 12-16. For metastatic colorectal cancer (mCRC), the relationship of the MMR/MSI phenotype and prognosis is unclear 17. Ben et al. found that CRC patients with the dMMR/MSI-H phenotype have a worse prognosis 18. Venderbosch et al. confirmed this result, but they suggested that the poor prognosis of dMMR appears to be driven by BRAF mutation status 19. However, studies by Goldstein and FUJIYOSHI et al. found that dMMR/MSI-H status has no correlation with prognosis 20, 21. For mCRC, only 5% of patients had the dMMR/MSI-H phenotype. One meta-analysis included studies showed that the effect of chemotherapy in mCRC patients with MSI status was similar to that of patients with MSS status 22. Another meta-analysis suggested that MSI-H status was beneficial to disease-free survival (DFS) of mCRC patients, whereas it was not beneficial to overall survival (OS)^23^. Recently, KEYNOTE-177 published the results dMMR/MSI-H mCRC patients received pembrolizumab or chemotherapy. The results showed the ORR of dMMR/MSI-H mCRC patients received chemotherapy was 33.1%, and the progression-free survival (PFS) was 8.2 months 24. Therefore, the aim of this study was to clarify in the real-world the PFS of dMMR/MSI-H mCRC patients who received first-line palliative chemotherapy.

## Materials and methods

### Patients

This study enrolled patients with mCRC who were diagnosed and received treatment in two Chinese centers, Sun Yat-sen University Cancer Center and the Second Affiliated Hospital of Kunming Medical University, between June 2010 and August 2019. The inclusion criteria were histologically proven mCRC with clear MMR or MSI status, and the exclusion criteria were incomplete clinicopathological or treatment information. The study was approved by the Institutional Research Ethics Committee of Sun Yat-sen University Cancer Center, and due to the retrospective nature of the study, consent from individual patients was not required.

### Data collection

Most of the data used in this study were extracted from the Bigdata Alliance for Colorectal Cancer (BACC) platform (YiduCloud Technology Ltd., Beijing, China) and were checked by the researchers. The BACC platform is a big-data intelligence platform that integrates multisource heterogeneous electronic health-records data from hospitals all over China. We collected the following data from mCRC patients via the platform: demographic and epidemiological information (age, gender, smoking, family history, etc.), date of diagnosis, histopathological grading, primary tumor site, synchronous metastasis or metachronous metastasis, metastatic site, *RAS*/*BRAF* status, MMR/MSI status, chemotherapy regimens, best overall response to chemotherapy, start time and progressive time of chemotherapy, and last follow-up time or date of death. We excluded patients who had incomplete clinicopathological; who had missing or invalid information about their efficacy or PFS of first-line palliative chemotherapy. For first-line palliative chemotherapy, the censored time of follow-up was 36 months. For second-line palliative chemotherapy, the censored time of follow-up was 24 months. Local treatment including resection, radiotherapy and interventional therapy of the metastasis.

MMR status was detected by immunohistochemistry, and dMMR was defined as the loss of one or more of the following proteins: MLH1, MSH2, MSH6, or PMS2. MSI status was detected by PCR molecular detection or next-generation sequencing (NGS), and MSI-H was defined as instability in two or more of the gene loci (or > 30%), MSI-L as instability in only one gene locus (or < 30%) and MSS as no gene locus instability. In this study, MSI-L was grouped with MSS.

### Statistical analysis

All statistical analyses were performed using R version 3.6.3 (http://www.r-project.org). The characteristics of patients were compared and evaluated by the Chi-square test.

Propensity scores were calculated in R Studio version 3.6.3 using a multivariable logistic regression model. The model included the following variables: age, CRC family history, metachronous or synchronous metastasis, primary tumor site, pathological differentiation and local treatment. The gene status of *NRAS, KRAS* and* BRAF* were not included in the model because they were unknown in more than half of the patients. In the PSM analysis, patients in the dMMR/MSI-H group were matched at a 1:4 ratios with those in the pMMR/MSS group. After assuring the comparability of the groups, treatment outcomes of first-line and second-line chemotherapy were compared between the dMMR/MSI-H and pMMR/MSS groups. The Kaplan-Meier method was used to estimate PFS and OS, and the log-rank test was used to compare PFS and OS. A two-sided *p* value and *p* value for the interaction of less than 0.05 was considered to indicate statistical significance.

## Results

### Comparison of the efficacy of first-line chemotherapy in the dMMR/MSI-H and pMMR/MSS populations

First, we found out 253 dMMR/MSI-H CRC patients from the BACC big data platform, and collected their detailed medical data. Then we excluded 87 patients with early stage or locally advanced tumor, 5 patients with incomplete clinicopathological information, 14 patients who did not receive chemotherapy, 70 patients who lacked complete first-line palliative treatment information, and there were remaining 77 dMMR/MSI-H mCRC patients with intact baseline and treatment information. Using the same method, we collected 510 pMMR/MSS mCRC patients who received first-line palliative treatment and had complete treatment information. We enrolled the eligible 77 dMMR/MSI-H and 510 pMMR/MSS mCRC patients for PMS analysis. After adjustment for clinicopathological characteristics, 71 dMMR/MSI-H and 254 pMMR/MSS patients were matched. Patient characteristics in the overall population and PSM cohort are summarized in Table 1. In the overall population, dMMR/MSI-H was associated with younger age, a positive family history, metachronous metastasis, right-sided primary tumor, poorly differentiated adenocarcinoma. In the PSM cohort, there were no differences of these variables between dMMR/MSI-H and pMMR/MSS patients.

As shown in Table 2, there was no significant difference in their choice of local treatment, chemotherapy regimens, and targeted therapy regimens between two groups. The ORR of pMMR/MSS mCRC patients was 35.4% (95%CI, 29.6%-41.7%), similar with that of dMMR/MSI-H mCRC patients (35.2%; 95%CI, 24.5%-47.5%; *p* = 1.00). The disease control rate (DCR) of pMMR/MSS mCRC patients was similar with that of dMMR/MSI-H mCRC patients so (74.4% vs 73.2%; *p* = 0.96).

**Figure 1:**
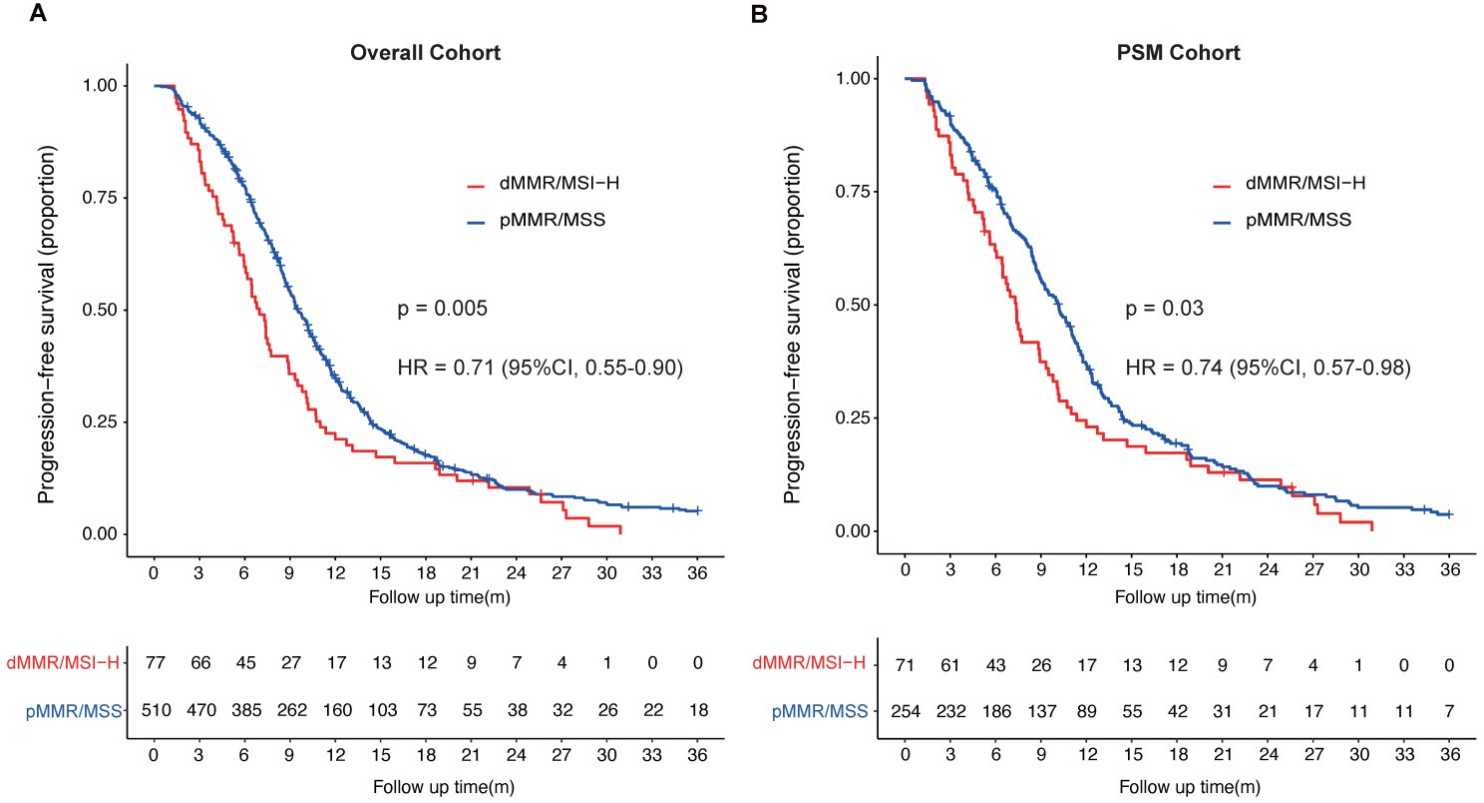
** Progression-free survival (PFS) of patients with metastatic colorectal cancer stratified by MMR or microsatellite status. A,** PFS of first-line palliative chemotherapy of mCRC patients (overall cohort) with different MMR or microsatellite status; **B,** PFS of first-line palliative chemotherapy of mCRC patients (PSM cohort) with different MMR or microsatellite status.

In overall cohort, Patients with dMMR/MSI-H had a median PFS of 7.0 months (95%CI, 6.0-8.9 months), significantly shorter than that of pMMR/MSS patients, who had a median PFS of 9.5 months (95%CI, 9.0-10.2 months) (HR = 0.71; 95%CI, 0.55-0.90; *p* = 0.005; Fig. 1A). In PSM cohort, the median PFS of dMMR/MSI-H patients was 7.4 months (95%CI, 6.4-9.3 months), of pMMR/MSS patients was 10.2 months (95%CI, 9.0-11.1 months) (HR = 0.74; 95%CI, 0.57-0.98; *p* = 0.03) (Fig. 1B). The univariable and multivariate cox analysis of overall cohort showed pathological differentiation poorly (HR = 1.30; 95%CI, 1.08-1.56), received local treatment (HR = 0.78; 95%CI, 0.65-0.94) and pMMR/MSS (HR = 0.76; 95%CI, 0.59-0.99) were the independent prognostic factors for the PFS of first-line palliative chemotherapy in mCRC patients (Table 3). What's more, the MMR/microsatellite status was still the independent prognostic factor for the PFS of first-line palliative chemotherapy in PSM cohort (pMMR/MSS; HR = 0.74; 95%CI, 0.57-0.98). The multivariate cox analysis of PSM cohort also found with hepatic metastasis (HR = 1.62; 95%CI, 1.27-2.08) and received local treatment (HR = 0.61; 95%CI, 0.47-0.78) were independent prognostic factors (Table 4).

**Figure 2:**
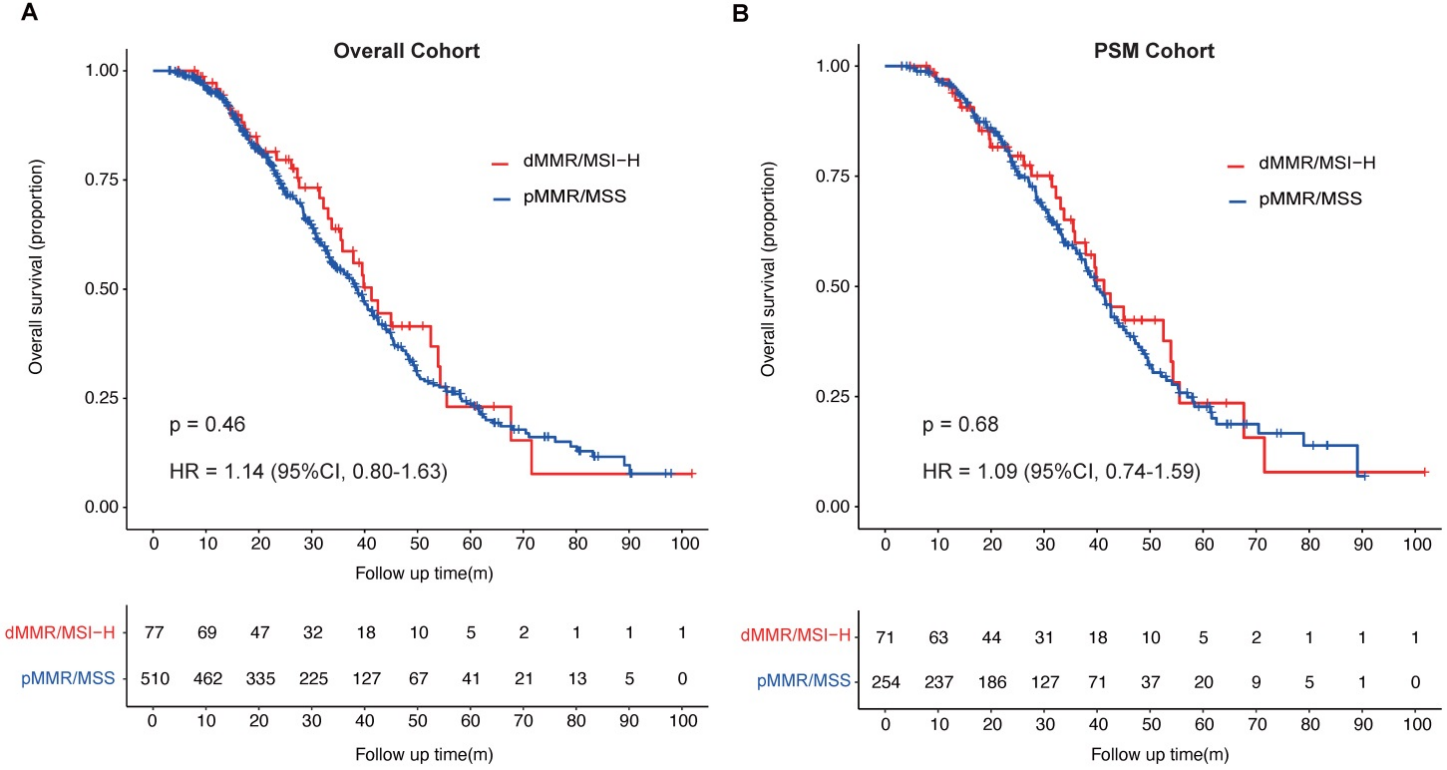
** Overall survival (OS) of patients with metastatic colorectal cancer stratified by MMR or microsatellite status. A,** OS of mCRC patients (overall cohort) with different MMR or microsatellite status; **B,** OS of mCRC patients (PSM cohort) with different MMR or microsatellite status.

Patients with pMMR/MSS in overall cohort had a median OS of 38.6 months (95%CI, 34.6-41.5 months), which was shorter than that of patients with dMMR/MSI-H in overall cohort, who had a median OS of 41.3 months (95%CI, 35.5-54.3 months). In PSM cohort, the median OS of patients with pMMR/MSS was 40.0 months (95%CI, 37.0-43.9 months), which was shorter than that of patients with dMMR/MSI-H (41.3 months, 95%CI, 35.5-55.5 months). There was no significant difference of the median OS between the two groups whether in overall cohort or in PSM cohort (overall cohort: HR = 1.14; 95%CI, 0.80-1.63; *p* = 0.46; PSM cohort: HR = 1.09; 95%CI, 0.74-1.59; *p* = 0.68; Fig. 2A & B).

### Comparison of the efficacy of second-line chemotherapy in the dMMR/MSI-H and pMMR/MSS populations

We enrolled the eligible 34 dMMR/MSI-H and 280 pMMR/MSS mCRC patients who received second-line chemotherapy for PMS analysis. After adjustment for clinicopathological characteristics, 33 dMMR/MSI-H and 117 pMMR/MSS patients were matched. Patient characteristics in the overall population and PSM cohort are summarized in Table S2. In the PSM cohort, there were no differences of clinicopathological characteristics between dMMR/MSI-H and pMMR/MSS patients.

For the two groups of patients, there was no difference in their choice of local treatment, chemotherapy or targeted therapy regimens. The ORR and DCR of second-line chemotherapy of dMMR/MSI-H mCRC patients was 9.1% and 66.7%, similar with that of pMMR/MSS mCRC patients (15.4% and 64.1%; *p* = 0.53 and *p* = 0.95) (Table S3). In overall cohort, median PFS was 5.4 months (95%CI, 3.8-7.9 months) in patients with dMMR/MSI-H tumors, and 5.4 months (95%CI, 4.8- 6.1 months) in patients with pMMR/MSS tumors, which was not significantly different (HR = 0.94; 95%CI, 0.65-1.39; *p* = 0.78). In PSM cohort, the median PFS of second-line chemotherapy was also no significantly difference (dMMR/MSI-H vs pMMR/MSS = 5.4 months [95%CI, 3.8-7.9 months] vs 5.7 months [95%CI, 4.9-7.7 months]; HR = 0.88; 95%CI, 0.59-1.33; *p* = 0.56). The median OS of second-line chemotherapy had no significantly different in overall cohort (dMMR/MSI-H vs pMMR/MSS = 31.7 months [95%CI, 31.0 months -NR] vs 26.7 months [95%CI, 24.8 vs 29.8 months]; HR = 1.37; 95%CI, 0.90-2.33; *p* = 0.25) or PSM cohort (dMMR/MSI-H vs pMMR/MSS = 31.7 months [95%CI, 31.0 months -NR] vs 33.7 [95%CI, 27.7-44.4 months]; HR = 0.98; 95%CI, 0.55-1.77; *p* = 0.96).

### Comparison of oxaliplatin-based versus irinotecan-based chemotherapy in the dMMR/MSI-H population

While compared the oxaliplatin-based versus irinotecan-based first-line chemotherapy in the dMMR/MSI-H population, we enrolled all 77 eligible patients. The ORR and DCR of dMMR/MSI-H patients who received irinotecan-based first-line chemotherapy were 54.5% (95%CI, 24.6%-81.9%) and 90.9% (95%CI, 57.1%-99.5%), respectively. For dMMR/MSI-H patients who received oxaliplatin-based first-line chemotherapy the ORR and DCR were 28.8% (95%CI, 18.1%-42.3%) and 67.8% (95%CI, 54.2%-79.0%), respectively. There were no significant differences in the ORR and DCR among patients who received different chemotherapies (*p* = 0.16, *p* = 0.16, respectively).

Patients with dMMR/MSI-H who received irinotecan had a median PFS of 7.6 months (95%CI, 3.4-10.7 months), which was longer than that of patients with dMMR/MSI-H who received oxaliplatin (6.7 months, 95%CI, 5.6-8.8 months) but not significantly so (HR = 0.87; 95%CI, 0.44-1.72; *p* = 0.69). The median OS of patients with dMMR/MSI-H status who received irinotecan in first-line palliative chemotherapy was 33.1 months (95%CI, 12.7 months-NR). For dMMR/MSI-H patients who received oxaliplatin in first-line palliative chemotherapy, the median OS was 45.0 months (95%CI, 35.8-54.3 months). There was no difference between them (HR = 0.65; 95%CI, 0.26-1.63; *p* = 0.35).

### Comparison of Cetuximab versus Bevacizumab in the dMMR/MSI-H population

While compared the cetuximab-containing versus bevacizumab-containing first-line treatment in the dMMR/MSI-H population, we enrolled all 77 eligible patients. For mCRC patients with a dMMR/MSI-H tumor, the ORR and DCR of patients who received bevacizumab combined with chemotherapy in palliative first-line chemotherapy were 55.0% (95%CI, 32.0%-76.2%) and 90.0%, respectively. The ORR and DCR of patients who received cetuximab in combination with chemotherapy were 44.4% (95%CI, 20.5%-56.1%) and 77.8%, respectively. Whether bevacizumab or cetuximab was combined with chemotherapy, both the ORR and DCR were higher than those who were treated with chemotherapy alone (ORR 22.2%, DCR 62.2%). However, only the difference in ORR between patients who received bevacizumab and those who did not was significant (*p* = 0.02).

Patients who received bevacizumab or cetuximab combined with chemotherapy had median PFS of 7.4 months (95%CI, 4.6-13.1 months) and 6.4 months (95%CI, 3.1-11.4 months), respectively. The PFS was no significant difference between patients received targeted therapy combined chemotherapy and chemotherapy only (*p* > 0.05). The median OS of patients who received bevacizumab and cetuximab was 31.5 months (95%CI, 19.8 months-NR) and 67.7 months (95%CI, 14.2 months-NR), respectively. The OS did not significantly differ between patients who received target therapy combined with chemotherapy and those who received chemotherapy alone (*p* > 0.05).

## Discussion

Our retrospective study presents a retrospective cohort of dMMR/MSI-H mCRC patients from two Chinese hospitals, with the purpose of exploring tumor prognosis and evaluating its sensitivity to chemotherapy and target therapy in real-world. We compared the clinicopathological characteristics of 77 dMMR/MSI-H mCRC patients with 510 pMMR/MSS mCRC patients who received systematic palliative treatment contemporaneously. According to our analysis, patients with dMMR/MSI-H mCRC tended to have higher rate of younger age, a positive family history, metachronous metastasis, right-side primary tumor, and poorly histology. These characteristics were consistent with previously published studies 18, 25-27.

In our study, median PFS of dMMR/MSI-H mCRC patients treated with first-line chemotherapy was 7.0 months in overall cohort and 7.4 in PSM cohort, which was similar to that observed in other studies but significantly shorter than that of pMMR/MSS patients in overall cohort (HR = 0.71; 95%CI, 0.55-0.90) or PSM cohort (HR = 0.74; 95%CI, 0.57-0.98). The median OS (38.6 months in overall cohort and 41.3 in PSM cohort) of dMMR/MSI-H mCRC patients was longer than that observed in other studies. Noticing that the OS of patients with pMMR/MSS tumor in our studies was also longer than that in similar studies, we suggested the long survival time might be caused by the high proportion (over 35%) of patients received local treatment in our centers. Patients with dMMR/MSI-H phenotype had shorter first-line PFS and longer OS than that with pMMR/MSS phenotype, which might be caused by the option of immunotherapy in subsequent treatment. Considering that the median OS of dMMR/MSI-H mCRC patients was similar to that of pMMR/MSS patients, our results and those from a recent large study contrast with those from some smaller studies on dMMR/MSI-H mCRC, suggesting that MMR status is not a prognostic factor for mCRC patients 20, 28, 29.

Whether dMMR/MSI-H is a predictive factor for chemotherapy is also controversial. After adjusted the unbalanced baseline characteristics, we compared the chemosensitivity of 71 dMMR/MSI-H mCRC patients to that of 254 matched pMMR/MSS patients. On the basis of their medical record, there was no significant difference in the first-line chemotherapy/target therapy choices of the two groups of patients. However, the first-line median PFS of dMMR/MSI-H patients was shorter than that of pMMR/MSS patients, and was shorter than KEYNOTE-177 (8.2 months), it may because the usage of targeted therapy less than 40% in our study^24^. The ORR and DCR of PSM cohort were similar, and ORR of our study was similar with KEYNOTE-177. Interestingly, in the second-line setting, two groups of patients exhibited numerically higher ORR, similar DCR and PFS, which indicated that, in the second-line setting, the chemosensitivity of dMMR/MSI-H patients might be better than or close to that of the pMMR/MSS mCRC patients. We noticed that nearly 80% of patients received oxaliplatin as the first-line therapy and irinotecan-based chemotherapy as the second-line palliative treatment in our study. Our result was consistent with the analysis of Alexandra et al^30^, whose study suggested that, compared with pMMR mCRC patients, patients with dMMR (n=27) had numerically lower RR (28.6% vs. 11.7%) in the first-line oxaliplatin-based treatment, and similar RR (7.4% versus 5.5%) in the second-line irinotecan-based treatment. Several studies have reported that, compared to pMMR/MSS patients, dMMR/MSI-H CRC patients were more sensitive to irinotecan, displaying a higher RR, DCR, and longer PFS 31, 32. Jeong Eun Kim 33 evaluated the efficacy of irinotecan‐containing chemotherapy for mCRC and found that the ORR, PFS, and OS of patients with dMMR were higher than those of patients with pMMR, but the differences were not statistically significant. When referred to oxaliplatin-based treatment, Müller reported that patients with MSI-H mCRC had a lower response rate to the CAPOX or FUFOX regimen in comparison with MSS patients 29, suggesting that dMMR status was predictive of resistance to oxaliplatin-based chemotherapy. However, more studies have found that MMR status does not significantly influence the ORR, PFS and OS of patients who receive oxaliplatin-based chemotherapy 22, 34, 35. The efficacy of different chemotherapy regimens for dMMR/MSI-H mCRC patients is also worth discussing. Our study indicated that dMMR/MSI-H mCRC patients who received first-line irinotecan-based chemotherapy had a higher ORR and PFS compared to those who received first-line oxaliplatin-based chemotherapy, but these differences were not statistically significant. Tougeron 36 observed significantly longer PFS and a trend for longer OS for irinotecan-based chemotherapy compared to oxaliplatin-based chemotherapy. However, J. Goldstein found that in mCRC with MSI-H, no significant differences in RR, PFS or OS were found between oxaliplatin- based and irinotecan-based chemotherapy 20.

Few studies have compared the sensitivity of target therapy for dMMR/MSI mCRC with that of target therapy for pMMR/MSS mCRC. Seung Tae Kim reported that when treated with cetuximab-containing or bevacizumab-containing chemotherapy, there was no significant difference between the MSI-H and MSS mCRC groups in treatment efficacy in either RR, DCR or PFS 37, 38. Kay Pogue-Geile 39 found from a post-hoc analysis of the NSABP C-08 study that patients diagnosed with dMMR colon cancer derived a significant survival benefit from bevacizumab in comparison to patients with a pMMR tumor. In our study, dMMR/MSI-H and pMMR/MSS mCRC patients showed similar RR and DCR in response to first-line cetuximab- or bevacizumab-based chemotherapy. However, PFS of pMMR/MSS patients was significantly longer than that of MSI-H patients. While the differences in treatment efficacy of different target therapies for dMMR/MSI-H tumor have been examined, there is no clear consensus to date. Yue Yu 40 reported that, compared with a chemotherapy-alone group, the bevacizumab treatment group achieved significantly longer PFS and a tendency to exhibit a higher ORR, whereas the cetuximab combined group did not. A post-hoc analysis of the CALGB/SWOG 80405 study also suggested that bevacizumab-containing chemotherapy conferred a survival advantage over cetuximab-containing chemotherapy 41. However, another later published study of a large cohort of non-selective dMMR patients 36 found there was no significant difference in OS between the anti-VEGF-containing and anti-EGFR-containing treatment groups. We found that the use of bevacizumab could result in a significantly higher ORR for dMMR/MSI-H mCRC patients, whereas cetuximab could not, but there were no differences in PFS and OS between the chemotherapy-alone group and the combined target therapy group.

The main limitation of our study is its retrospective design, which may to some degree influence its external validity. Moreover, because of the low incidence, the number of dMMR/MSI-H patients in the subgroup analysis was relatively small, which made it difficult to detect significant differences between the outcomes of different treatments among these patients.

In conclusion, our study showed that the efficacy and survival of dMMR/MSI-H mCRC patients received first-line palliative chemotherapy in real world. The status of MMR/microsatellite was not a prognostic factor for mCRC patients, but it was associated with shorter PFS in first-line palliative chemotherapy.

## Supplementary Material

Supplementary tables.

## Figures and Tables

**Table 1:** Characteristics of mCRC patients with different MMR or microsatellite status (first-line palliative chemotherapy): overall and propensity score-matched cohorts

Features	Overall cohort (n = 587)			PSM cohort (n = 325)		
	pMMR/MSS (n=510)	dMMR/MSI-H (n=77)	*p* ^†^	pMMR/MSS (n=254)	dMMR/MSI-H (n=71)	*p* ^†^
**Age, n (%)**			0.03			0.64
<60	350 (68.6)	63 (81.8)		195 (76.8)	57 (80.3)	
≥60	160 (31.4)	14 (18.2)		59 (23.2)	14 (19.7)	
**Sex, n (%)**			0.21			0.15
Female	194 (38.0)	23 (29.9)		101 (39.8)	21 (29.6)	
Male	316 (62.0)	54 (70.1)		153 (60.2)	50 (70.4)	
**CRC family history, n (%)**			0.08			0.37
No	481 (94.3)	68 (88.3)		233 (91.7)	62 (87.3)	
Yes	29 (5.7)	9 (11.7)		21 (8.3)	9 (12.7)	
**Smoking, n (%)**			0.87			0.88
No	369 (72.4)	57 (74.0)		185 (72.8)	53 (74.6)	
Yes	141 (27.6)	20 (26.0)		69 (27.2)	18 (25.4)	
**Time of Metastasis, n (%)**			<0.001			0.33
Metachronous metastasis	130 (25.5)	38 (49.4)		96 (37.8)	32 (45.1)	
Synchronous metastasis	380 (74.5)	39 (50.6)		158 (62.2)	39 (54.9)	
**Primary tumor site, n (%)**			0.001			0.55
Left	372 (72.9)	41 (53.2)		159 (62.6)	41 (57.7)	
Right	138 (27.1)	36 (46.8)		95 (37.4)	30 (42.3)	
**Pathological differentiation, n (%)**			0.01			0.88
Moderately	317 (62.2)	36 (46.8)		124 (48.8)	36 (50.7)	
Poorly	193 (37.8)	41 (53.2)		130 (51.2)	35 (49.3)	
**Hepatic metastasis, n (%)**			0.75			0.50
No	205 (40.2)	33 (42.9)		121 (47.6)	30 (42.3)	
Yes	305 (59.8)	44 (57.1)		133 (52.4)	41 (57.7)	
**Pulmonary metastasis, n (%)**			0.64			1.00
No	340 (66.7)	54 (70.1)		177 (69.7)	50 (70.4)	
Yes	170 (33.3)	23 (29.9)		77 (30.3)	21 (29.6)	
**Distant lymph node metastasis, n (%)**			0.91			0.78
No	364 (71.4)	56 (72.7)		183 (72.0)	53 (74.6)	
Yes	146 (28.6)	21 (27.3)		71 (28.0)	18 (25.4)	
**Peritoneum metastasis, n (%)**			0.49			1.00
No	374 (73.3)	53 (68.8)		169 (66.5)	47 (66.2)	
Yes	136 (26.7)	24 (31.2)		85 (33.5)	24 (33.8)	
**NRAS, n (%)**			<0.001			<0.001
Mutation	14 (2.7)	9 (11.7)		6 (2.4)	5 (7.0)	
Unknown	241 (47.3)	52 (67.5)		121 (47.6)	50 (70.4)	
Wild	255 (50.0)	16 (20.8)		127 (50.0)	16 (22.5)	
**KRAS, n (%)**			0.034			0.13
Mutation	120 (23.5)	18 (23.4)		58 (22.8)	14 (19.7)	
Unknown	187 (36.7)	39 (50.6)		99 (39.0)	37 (52.1)	
Wild	203 (39.8)	20 (26.0)		97 (38.2)	20 (28.2)	
**BRAF, n (%)**			<0.001			<0.001
Mutation	34 (6.7)	9 (11.7)		18 (7.1)	5 (7.0)	
Unknown	216 (42.4)	51 (66.2)		111 (43.7)	49 (69.0)	
Wild	260 (51.0)	17 (22.1)		125 (49.2)	17 (23.9)	

Abbreviations: CRC: colorectal cancer; ^†^ Pearson's Chi-square test.

**Table 2:** First-line palliative chemotherapy in propensity score-matched cohort

Feature	pMMR/MSS (n=254)	dMMR/MSI-H (n=71)	*p* ^†^
**Chemotherapy, n (%)**			0.67
FOLFOX	119 (46.9)	29 (40.8)	
FOLFIRI^a^	33 (13.0)	10 (14.1)	
FOLFOXIRI	9 (3.5)	1 (1.4)	
XELOX	75 (29.5)	26 (36.6)	
XELODA	10 (3.9)	4 (5.6)	
Other	8 (3.1)	1 (1.4)	
**Chemotherapeutics, n (%)**			0.99
Oxaliplatin-based	195 (76.8)	55 (77.5)	
Irinotecan-based^a^	34 (13.4)	9 (12.7)	
Both Oxaliplatin and Irinotecan	12 (4.7)	3 (4.2)	
Other	13 (5.1)	4 (5.6)	
**Targeted therapy, n (%)**			0.87
Bevacizumab or cetuximab containing	98 (38.6)	26 (36.6)	
Other	156 (61.4)	45 (63.4)	
**Local treatment, n (%)**			0.94
No	168 (66.1)	46 (64.8)	
Yes	86 (33.9)	25 (35.2)	
**Efficacy, n (%)**			
ORR	90 (35.4)	25 (35.2)	1.00
DCR	189 (74.4)	52 (73.2)	0.96

Abbreviations: DCR: disease control rate; FOLFIRI: irinotecan, fluorouracil and calcium folinate; FOLFOX: oxaliplatin, fluorouracil and calcium folinate; FOLFOXIRI: oxaliplatin, irinotecan, fluorouracil and calcium folinate; ORR: objective response rate; XELODA: capecitabine; XELOX: oxaliplatin and capecitabine. ^†^ Pearson's Chi-square test; ^a^ One patient used FOLFOXIRI only twice, then stop using oxaliplatin.

**Table 3:** The univariate and multivariate analysis of overall cohort

Variable	Univariable Cox analysis		Multivariate Cox analysis	
	HR (95%CI)	p value	HR (95%CI)	p value
**Age**				
<60	Ref	0.69		
≥60	0.96 (0.8-1.16)			
**Sex**				
Female	Ref	0.97		
Male	1 (0.83-1.19)			
**CRC family history**				
No	Ref	0.28		
Yes	1.21 (0.85-1.73)			
**Smoking**				
No	Ref	0.23		
Yes	0.89 (0.73-1.08)			
**Time of Metastasis**				
Metachronous metastasis	Ref	0.023		0.14
Synchronous metastasis	0.80 (0.66-0.97)		0.86 (0.70-1.05)	
**Primary tumor site**				
Left	Ref	0.19		
Right	1.13 (0.94-1.37)			
**Pathological differentiation**				
Moderately	Ref	<0.001		0.005
Poorly	1.39 (1.17-1.66)		1.30 (1.08-1.56)	
**Hepatic metastasis**				
No	Ref	0.056		
Yes	1.19 (1-1.42)			
**Pulmonary metastasis**				
No	Ref	0.77		
Yes	1.05 (0.87-1.27)			
**Distant lymph node metastasis**				
No	Ref	0.31		
Yes	1.11 (0.91-1.34)			
**Peritoneum metastasis**				
No	Ref	0.64		
Yes	1.05 (0.87-1.27)			
NRAS*				
Mutation	Ref	0.008		
Unknown	0.50 (0.32-0.78)			
Wild	0.57 (0.36-0.89)			
KRAS*				
Mutation	Ref	0.07		
Unknown	0.83 (0.66-1.04)			
Wild	1.03 (0.82-1.29)			
BRAF*				
Mutation	Ref	<0.001		
Unknown	0.52 (0.38-0.73)			
Wild	0.56 (0.40-0.78)			
**Chemotherapy**				
FOLFOX	Ref	0.16		
FOLFIRI^a^	1.16 (0.87-1.54)			
FOLFOXIRI	1.24 (0.72-2.13)			
XELOX	1.03 (0.84-1.25)			
XELODA	1.09 (0.68-1.76)			
Other	2.17 (1.21-3.88)			
**Chemotherapeutics**				
Oxaliplatin-based	Ref	0.44		
Irinotecan-based	1.18 (0.9-1.54)			
Both Oxaliplatin and Irinotecan	1.25 (0.81-1.94)			
Other	1.20 (0.78-1.84)			
**Targeted therapy**				
Bevacizumab or cetuximab containing	Ref	0.15		
Other	1.14 (0.955-1.36)			
**Local treatment**				
No	Ref	<0.001		
Yes	0.73 (0.61-0.87)		0.78 (0.65-0.94)	0.009
**MMR/microsatellite status**				
dMMR/MSI-H	Ref	0.0054		
pMMR/MSS	0.71 (0.55-0.90)		0.76 (0.59-0.99)	0.04

*Because of missing value (>30%) KRAS, NRAS and BRAF were not included in the final multivariable model.

**Table 4:** The univariate and multivariate analysis of PSM cohort

Variable	Univariable Cox analysis		Multivariate Cox analysis	
	HR (95%CI)	p value	HR (95%CI)	p value
**Age**				
<60	Ref	0.63		
≥60	0.935 (0.71-1.23)			
**Sex**				
Female	Ref	0.87		
Male	1.02 (0.81-1.29)			
**CRC family history**				
No	Ref	0.26		
Yes	1.27 (0.84-1.91)			
**Smoking**				
No	Ref	0.43		
Yes	0.90 (0.7-1.17)			
**Time of Metastasis**				
Metachronous metastasis	Ref	0.12		
Synchronous metastasis	0.83 (0.66-1.05)			
**Primary tumor site**				
Left	Ref	0.93		
Right	1 (0.78-1.25)			
**Pathological differentiation**				
Moderately	Ref	0.07		
Poorly	1.24 (0.99-1.56)			
**Hepatic metastasis**				
No	Ref	0.006		
Yes	1.38 (1.09-1.73)		1.62 (1.27-2.08)	<0.001
**Pulmonary metastasis**				
No	Ref	0.95		
Yes	1.01 (0.79-1.29)			
**Distant lymph node metastasis**				
No	Ref	0.08		
Yes	1.26 (0.98-1.63)			
**Peritoneum metastasis**				
No	Ref	0.85		
Yes	1.02 (0.81-1.3)			
**Chemotherapy**				
FOLFOX	Ref	0.48		
FOLFIRI^a^	1.11 (0.78-1.57)			
FOLFOXIRI	1.54 (0.81-2.93)			
XELOX	1.03 (0.79-1.34)			
XELODA	0.95 (0.55-1.66)			
Other	0.91 (0.97-3.77)			
**Chemotherapeutics**				
Oxaliplatin-based	Ref	0.54		
Irinotecan-based	1.11 (0.80-1.54)			
Both Oxaliplatin and Irinotecan	1.48 (0.87-2.49)			
Other	1.05 (0.64-1.72)			
**Targeted therapy**				
Bevacizumab or cetuximab containing	Ref	0.1		
Other	1.22 (0.97-1.55)			
**Local treatment**				
No	Ref	0.01		
Yes	0.73 (0.58-0.93)		0.61 (0.47-0.78)	<0.001
**MMR/microsatellite status**				
dMMR/MSI-H	Ref	0.03		
pMMR/MSS	0.74 (0.57-0.98)		0.74 (0.57-0.98)	0.03

## Abbreviations

MMR: Mismatch repair
dMMR: Deficient mismatch repair
MSI: Microsatellite instability
mCRC: Metastatic colorectal cancer
pMMR: Proficient mismatch repair
MSS: Microsatellite stable
PSM: Propensity score matching
ORR: Objective response rate
PFS: Progression-free survival
OS: Overall survival
CRC: Colorectal cancer
MSI-H: High microsatellite instability
MSI-L: Low microsatellite instability
LS: Lynch syndrome
HNPCC: Hereditary nonpolyposis colorectal carcinoma
5-FU: 5-fluorouracil
DFS: Disease-free survival
NGS: Next-generation sequencing
DCR: Disease control rate
XELOX: Oxaliplatin and capecitabine
FOLFOX: Oxaliplatin, fluorouracil and calcium folinate
FOLFIRI: Irinotecan, fluorouracil and calcium folinate
FOLFOXIRI: Oxaliplatin, irinotecan, fluorouracil and calcium folinate
XELODA: Capecitabine
CPT-11: Irinotecan
